# Application of pancreatic phospholipase A2 for treatment of bovine mastitis

**DOI:** 10.1371/journal.pone.0203132

**Published:** 2018-08-27

**Authors:** Eyal Seroussi, Shlomo E. Blum, Oleg Krifucks, Yaniv Lavon, Gabriel Leitner

**Affiliations:** 1 Institute of Animal Science, Agricultural Research Organization (ARO), Rishon LeTsiyon, Israel; 2 National Mastitis Reference Center, Department of Bacteriology, Kimron Veterinary Institute, Bet Dagan, Israel; 3 Israel Cattle Breeders Association, Caesarea, Israel; University of Illinois, UNITED STATES

## Abstract

Recent findings have indicated that secreted phospholipases A2 (sPLA2s) have anti-inflammatory functions, including relief of symptoms in a mouse model of mastitis. This prompted us to investigate the therapeutic application of sPLA2, PLA2G1B, for bovine mastitis. Initial testing of PLA2G1B's effect on bovine mammary epithelial cell (bMEC) line PS revealed no changes in cell viability or cytokine-secretion pattern. However, when cells were first treated with lipopolysaccharide endotoxin (LPS) or live bacteria (*Escherichia coli* or *Staphylococcus aureus*), incubation with PLA2G1B significantly improved cell viability, suggesting involvement of sPLA2s in protecting membranes from lipid-peroxidation damage, rather than a bactericidal action. When PLA2G1B was applied simultaneously with LPS, a significant short-term reduction in interleukin-8 secretion was observed compared with bMECs treated only with LPS, supporting previous reports that PLA2G1B affects interleukin-8 signaling in similar cells. Following the favorable outcome of the *in vitro* experiments, we tested PLA2G1B *in vivo* by mammary infusion into infected glands. In one of a small sample (n = 4) of lactating cows chronically infected with *Streptococcus dysgalactiae*, a single PLA2G1B treatment completely cleared inflammation and bacteria, demonstrating its potential to cure subclinical mastitis. PLA2G1B treatment did not affect coagulase-negative staphylococci infection. These types of mastitis may involve formation of a resistant biofilm, and its elimination may relate to sPLA2s' characteristic ability to aggregate with cellular debris, facilitating their internalization by macrophages. In a bovine model of clinical mastitis based on introduction of *E*. *coli* via the streak canal, a single mammary infusion of PLA2G1B led to faster recovery to pre-infection milk-yield levels and decrease of somatic cell counts. In this case, all of sPLA2s' modes of resolving inflammation may apply, including competitive binding of the sPLA2s’ receptor, the inactivation of which confers resistance to endotoxic shock. Hence, this study strongly supports further research into PLA2G1B as a cure for bovine mastitis.

## Introduction

Mastitis is an inflammation of the mammary gland, caused by different bacteria, that is considered to be the costliest syndrome in the dairy farming [1]. Animal welfare standards require treating clinical mastitis during lactation, in most cases with antibiotics [2]-resulting in discarded milk due to the presence of abnormal milk and antibiotic residues, or by inducing involution of the infected gland or culling the cow. Moreover, antibiotic treatment is less effective against environmental coliforms and streptococci (other than *Streptococcus agalactiae*) and biofilm-forming pathogens [3,4] and in most cases of subclinical chronic infection; it is not used as the application to the supply chain management is on cost only. Consequently, alternative approaches, such as vaccination and/or approachable non-antibiotic medicines are sought and in the future, due to the potential development of antibiotic resistances, it is expected that alternative treatment measures and prevention will be more relevant than today.

The probability of developing mastitis involves complex interactions between the etiological agent and host genetic factors. Penetration of bacteria into the mammary gland through the streak canal, the major barrier to entry [5], is thought to be affected by both environmental and genetic factors. However, the effect of genetics leading to an easily penetrable morphology of the streak canal can be readily counteracted by sanitary measures and improved husbandry (i.e. use of modern milking machines capable of milking cows without injuring the udder) [6]. Pathogens are part of the microbiota in the cow environment and cow skin, including the external part of the streak canal. The anatomical and physiological components, a keratin plug coating the inlayer of the streak canal and contraction of the teat sphincter muscle closing the teat orifice between milkings, are the first lines of mammary defense [7]. Following bacterial invasion, the innate immunity factors and neutrophils are first to act against Gram-negative organisms by killing and phagocytosing the pathogens [8]. Glands infected with most types of Gram-positive bacteria garner a more gradual concentration of lymphocytes and regulatory T cells [9]. Initiation and propagation of the immunovascular inflammatory response are mediated by molecular mediators, of which lipid mediators have been shown to play an important role in dairy cattle [10]. Although it is impossible to know or calculate the percentage of bacterial elimination by the natural mechanisms of the mammary defense, most cows, most of the time, are not infected. Moreover, even under the worst management and environmental conditions, many cows remain healthy [7]. This implies that genetic factors are of primary importance in the probability of developing mastitis and indeed highly productive breeds are more susceptible to mastitis [11]. Indeed, genome-wide association studies have pointed to numerous quantitative trait loci for dairy mastitis. Nevertheless, only a few genes have been established as mastitis resistant genes, most of them play a role in presenting antigens to cells of the immune system such as those of the major histocompatibility complex and the Toll-like receptors [9]. The response to an infecting pathogen is dependent on the bacterial species, the host response and their interaction. The rate and efficiency of the innate immunity in recognizing and responding to the pathogen are key factors in infection duration and the severity of the damage to mammary tissues. Crucial to this response is the infiltration of leukocytes through the tight junctions between mammary epithelial cells (MECs). However, during active intramammary infection, permeability is increased as a result of both direct damage by bacterial toxins and the influence of pro-inflammatory mediators such as interleukin (IL) 1β, IL-6, IL-8, tumor necrosis factor alpha (TNFα), histamine and interferon γ, as well as complement components, lactoferrin, and other soluble proteins and enzymes [8,12,13].

Several lines of evidence indicate that mammalian secreted phospholipases A2 (sPLA2s) have a role in both pro- and anti-inflammatory processes [14]; however, their exact role in modulating infection, such as in sepsis, is still debatable [15]. Pro-inflammatory roles were originally assumed when these enzymes were first isolated and shown to release the key inflammatory intermediate arachidonic acid from cell-membrane phospholipids. However, recent studies suggest that they have primarily an anti-inflammatory role in the inflammatory response [16]. This potential role of sPLA2s in resolving inflammatory situations has been suggested to involve both catalytic and non-catalytic functions, in the following four suggested modes of action: (i) first suggested in the mid-1960s, a catalytic mechanism in which sPLA2s help resolve inflammation by protecting cell membranes from injury and contributing to phospholipid remodeling for membrane homeostasis [17,18]; (ii) another catalytic inflammation-resolving action is the antibacterial function of sPLA2s, best exemplified in human tears where PLA2G2A is the principal bactericide for staphylococci and other Gram-positive bacteria [19]; (iii) a non-catalytic function has been suggested to involve formation of supramolecular aggregates with anionic phospholipids in vesicles or debris, which facilitates their internalization by macrophages [16]; (iv) an alternative suggestion is that the non-catalytic anti-inflammatory action of sPLA2s involves competitive binding of the broad-range PLA2 receptor PLA2R1, simulating its deficiency, which is known to confer resistance to lethality in mice induced by lipopolysaccharide endotoxin (LPS) [20].

We have previously shown that non-bactericidal sPLA2s act as anti-inflammatory factors in the murine mammary gland and suggested them as candidate therapeutic agents for mastitis [20]. In cattle, the commercially available bovine PLA2G1B (PLA2 from bovine pancreas, cat. no. P8913, Sigma, Saint Louis, MO) is an attractive candidate for this purpose, being a stable and economically efficient PLA2. The objectives of this study were to (i) examine PLA2G1B-induced reduction of *Escherichia coli* and *Staphylococcus aureus* virulence *in vitro* in immortalized MECs; (ii) assess the usefulness and effectiveness of intramammary infusion of PLA2G1B as a treatment for cows with clinical or subclinical mastitis caused by different bacteria.

## Materials and methods

### Bovine mammary epithelial cells

The immortalized bovine MEC (bMEC) line PS [21], kindly provided by Dr. Pierre Germon, INRA, Infectiologie et Santé Publique, Nouzilly, France, was cultured in T-75 flasks (Corning Glass Works, Corning, NY) in growth medium consisting of advanced DMEM/F12 medium (Gibco Brl, Grand Island, NY) containing 4 ng/mL hydrocortisone (Gibco), 2 mM glutamine, 20 mM HEPES, 10 ng/mL insulin-like growth factor (IGF1-Rat), 5 ng/mL recombinant bovine fibroblast growth factor and 5 ng/mL epidermal growth factor (EGF-Mouse). Growth factors were obtained from Biological Industries (Bet Haemek, Israel). Bovine MECs were detached by EDTA/trypsin (Biological Industries), washed with Hanks Balanced Sole Solution (HBSS, Biological Industries) supplemented with 12.5% FBS (Biological Industries) and seeded in 24- to 48-well culture plates (Greiner, Germany) at 2 × 10^4^ cell/mL in growth media. The plates were incubated at 37°C in a CO_2_-incubator and after 24 h, growth medium were replaced with stimulation medium (SM) (growth medium without growth factors) overnight. In some experiments, SM were replaced with fresh SM containing 10 μg/mL LPS (Sigma) with or without PLA2G1B at a final concentration of 10 or 20 μg/mL. In other experiments, LPS or the bacteria *S*. *aureus* ZO3984 or *E*. *coli* VL2874 were added to a final concentration of 10^5^ CFU/mL for 4 h. In addition, incubated cells were washed with SM containing the antibiotics penicillin (200 μg/mL), streptomycin (0.1 mg/mL) and amphotericin 0.0025 mg/mL (Biological Industries) in HBSS and fresh SM with or without PLA2G1B (20 μg/mL) added to the wells. Cytokine production (TNFα, IL-1β, IL-8) was evaluated in cell supernatants following 24, 48 and 72 h incubation after removal of LPS or bacteria using commercial ELISA kits and according to the instructions of the manufacturer: Bovine ELISA TNFα kit (VetSet, Kingfisher Biotech., St. Paul, MN), Bovine IL-1β Elisa set (Thermo Fisher Scientific, Rockford, IL) and ELISA Assay for Bovine IL-8 (Mabtech, Naka Strand, Sweden), respectively. Viability of bMECs was determined with addition the fluorescent dye Alamar Blue (Bio-Rad, Oxford, UK). The plates were read with a GENios Plus plate reader (Tecan, Salzburg, Austria) with emission filter of 590 nm and excitation filter of 535 nm.

### Cultivation of bacteria

The following bacteria were prepared for inoculation: *E*.*coli* strain P4, isolated from acute mastitis [22] and widely used as a model strain in mastitis research, was obtained from NCIMB (catalogue no. 702070) and propagated once before storage; field-isolated *S*. *aureus* ZO3984 (β hemolytic) [23,24] and *E*. *coli* VL2874 were previously genotyped and analyzed by whole-genome sequencing and phenotyping [25,26,27]. Bacteria were recovered from stock (-80°C in brain heart infusion, 25% w/v glycerol) on blood agar (tryptose blood agar base; Becton-Dickinson, Sparks, MD, with 5% washed sheep erythrocytes), and incubated aerobically at 37°C overnight. Bacteria were harvested and washed in pyrogen-free saline (PFS). Bacterial concentration was determined by colony counting. For *in vivo* inoculation, bacteria were suspended in PFS and stored at 4°C for 10 h. Bacterial concentration was adjusted with PFS before challenge, aiming for an inoculum of about 30–100 CFU in 3 mL PFS. Final bacterial concentrations assessed from inoculum aliquots separated just prior to challenge ranged between 10 and 30 CFU per 3 mL PFS. For *in vitro* inoculation, bacteria were suspended at 10^5^ CFU/mL Roswell Park Memorial Institute (RPMI) media.

### Animals and study design

All treatment protocols were approved by the Institutional Animal Care Committee of the Agricultural Research Organization, The Volcani Center, Bet Dagan, Israel. This committee specifically approved this study (Decision # 16_b7736_10). Israeli Holstein cows of the research herd of the Volcani Center produced an average milk yield of >11,000 L per 305 lactation days. The dairy parlor was equipped with a computerized AfiFarm herd management system and AfiLab milk analyzer, providing online data on gross milk composition and conductivity. Cows were milked thrice daily. The study included healthy cows, in lactations 2–4, which were free of infection based on three consecutive bacteriological examinations and milk somatic cell score (SCC) <100,000 cell/mL, and cows naturally infected with *Streptococcus dysgalactiae* or coagulase-negative staphylococci (CNS) in a chronic phase (SCC >500,000 cell/mL for 2–3 months). Chronic phase was confirmed by bacteria isolation and identification of the same bacteria to the species level at least three times. In addition, healthy cows were challenged with *E*. *coli*. Animals were fed a typical Israeli mixed ration (65% concentrate and 35% forage, 17% protein, w/w) *ad libitum* in mangers located in sheds. The means of ages and weights of the sampled cows were 53.4 months (range: 45–67 months) and 633 kg (range: 469–880 kg), respectively. Three experiments were conducted.

#### Experiment 1

Healthy cows were used to study the mammary gland response to intramammary PLA2G1B infusion at different doses. Intramammary inoculation was performed aseptically after the morning milking. Teats were thoroughly cleaned, dried, disinfected with 7.5% povidone-iodine and wiped with PARASTERILE antiseptic cloth (Johnson Diversey Israel, Yavne, Israel). Two PLA2G1B doses (20 or 40 μg) were diluted in 5 mL PFS, and three cows were infused in one mammary-gland quarter per cow. PFS (5 mL) was also infused into the contralateral quarter. Cows were observed for symptoms of local inflammation for three days after the infusion. For bacteriological, milk-composition, SCC, and leukocyte-differentiation tests (% polymorphonuclear leukocytes-PMNs), milk was sampled before and after intramammary infusion (0, 4, 24 and 48 h).

#### Experiment 2 (observation)

Twelve cows that were 70–130 days in milk, producing >45 L/day, and that had chronically infected mammary glands (for ~60 days) were treated. Of these, four were infected with *S*. *dysgalactiae* in one gland, and eight were infected with CNS in one or two glands. Treatment consisted of infusing 30 μg PLA2G1B diluted in 20 mL PFS. Cows were infused once post-milking in the infected gland. Milk was sampled before infusion, and daily up to 10 days post-treatment for bacteriological, milk-composition, SCC, and leukocyte-differentiation tests.

#### Experiment 3

Twelve healthy cows were divided into pairs according to lactation, days in milk, daily milk yield, and SCC. All cows were challenged in one gland per cow post-milking with 30–100 CFU/gland of *E*. *coli* strain P4 in 3 mL PFS. At 24 h post-challenge, one cow per pair was treated with 30 μg PLA2G1B diluted in 20 mL PFS, and the second cow was not treated and served as a control. Development of clinical symptoms (edema and pain) was recorded during the first 24 h, including rectal temperature measurements every 4 h. For bacteriological, milk-composition, SCC, and leukocyte-differentiation tests, milk was sampled before and after infusion (0, 1, 2, 4, 7 and 17 days).

### Sampling procedures

For bacteriology, milk-composition, SCC, and leukocyte-differentiation tests (% polymorphonuclear leukocytes-PMNs), milk was sampled before and after intramammary infusion. Before milk sampling, teats were cleaned and disinfected, and the foremilk was discarded. For bacteriological tests, milk was collected aseptically (3 mL) into sterile tubes. For the other tests, the gland was milked into a separate container and milk volume was recorded, gently mixed and a sample of 0.5–1 L was collected for milk composition and SCC analysis (Fossomatic 360-Foss Electric, Hillerød, Denmark) at the Israel Cattle Breeders’ Association Laboratory (Caesarea, Israel). Leukocyte differentiation was tested by flow cytometry (FACScalibur, Becton-Dickinson, San Jose, CA) as previously described [28].

### Bacteriological analyses

Milk bacteriological identification was conducted according to the National Mastitis Council [29]. In experiment 2, 10 μL from each sample was spread on a blood agar plate (nutrient agar with 5% washed sheep erythrocytes) and on MacConkey agar plates for bacterial isolation. Bacteria were identified by classical bacteriological methods [29]. CNS were classified to genus-level, whereas streptococci were classified to species level with the API Rapid ID 32 STREP kit (bioMerieux, Marcy lEtoile, France), according to the manufacturer’s instructions. In experiment 3, milk samples were 10-fold serially diluted for enumeration of *E*. *coli* colonies on MacConkey agar; and *E*. *coli* representative colonies were confirmed to be of the same genotype as the inoculated strains by amplification of enterobacterial repetitive intergenic consensus sequences (ERIC-PCR [30]).

### Statistical analysis

The current study consisted of five different experiments. The first two experiments were performed *in vitro* using bMECs. The other three experiments were performed in the whole animal (*in vivo*). For the *in vitro* studies, two different models were used. Both analyses were carried out using the mixed procedure of SAS (SAS Institute, 2009) with the general form: result = group + time + group × time + error, where group = seven or eight groups of SM with different inclusions (control- no treatment, PLA2G1B, LPS, LPS+PLA2G1B, *E*. *coli*, *E*. *coli*+PLA2G1B, *S*. *aureus* and *S*. *aureus*+PLA2G1B), time = three measurement time points (24 h, 48 h, or 72 h). Rank transformation following Student’s *t*-test was performed to compare between groups challenged by the same inflammation agent with or without PLA2G1B.

For the *in vivo* studies, three different models were used. All analyses were carried out using the mixed procedure of SAS (SAS Institute, 2009). Experiments 1 and 3 had the general form: result = group + time + group × time + error, where group = three different groups (control, 20 and 40 μg/gland PLA2G1B), time = four (experiment 1; 0, 24 h, 48 h, or 72 h) or seven (experiment 3; 0, 1, 2, 3, 4, 7, 21 days after intramammary infusion) measurement time points. Experiment 2 had the general form: result = cow + time + error, where cow = four different cows (1–4), time = three different measurement time points post-inoculation (0, 5 and 10 days). Data are presented as means and standard error of the mean (SEM). Histograms and graphs were plotted with Clustered Column and Scatter Chart tools, respectively, with either straight or smooth line options (Excel 2007, Microsoft, Redmond, WA).

## Results

### *In vitro* bovine mammary epithelial cell treatments

**Fig 1:**
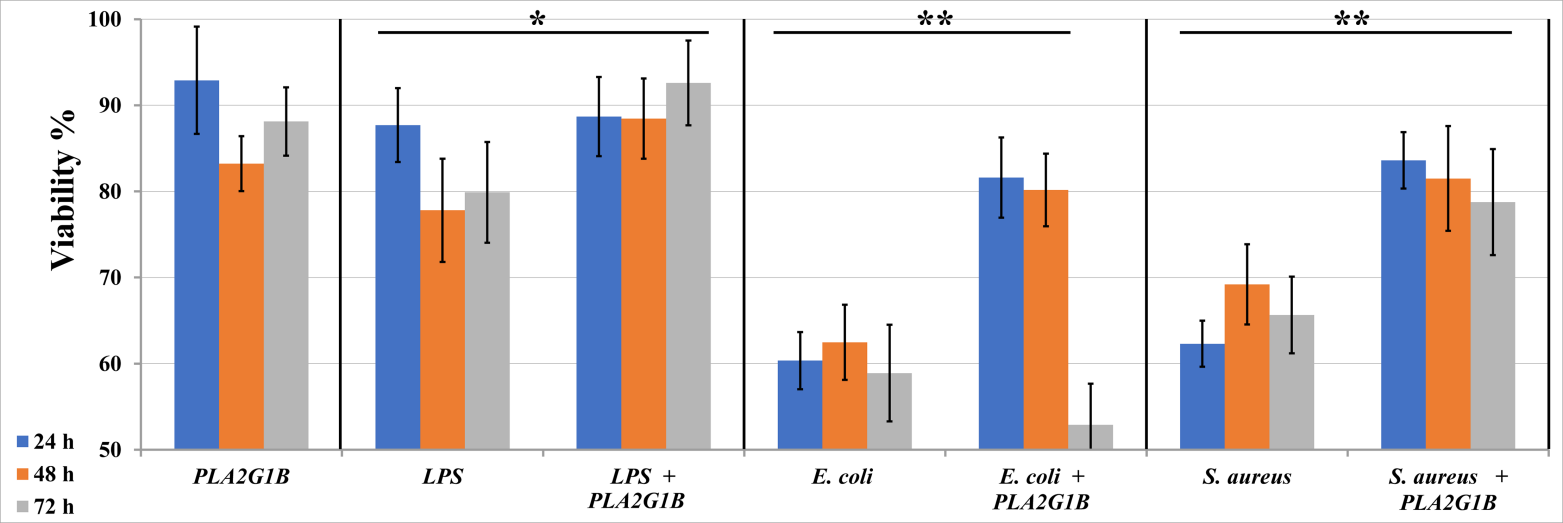
Effect of incubation with PLA2G1B (20 μg/mL) on viability of bovine mammary epithelial cells treated with LPS, *Escherichia coli* or *Staphylococcus aureus*. Cells were seeded in 48-well culture plates and examined at three time points post-treatment (1 day, blue; 2 days, orange; 3 days, gray). Cell viability was calculated as percentage of the initial count. Standard error bars are presented (n = 3). Asterisks denote a significant difference between the mean of means of the two groups of time points denoted by each of the horizontal bars (*P* < 0.05 by Student’s *t*-test, n = 6–9, *, **, with or without rank transformation, respectively).

To explore the safety and feasibility of applying PLA2G1B as a treatment for mastitis and considering cell viability as a criterion that reflects cellular damage, we first applied it to *in vitro*-cultured cells. Incubation of bMECs with 10 μg/mL PLA2G1B did not cause any changes in cell viability, which was ~90% of the initial count (Fig 1). Treatment of bMECs with a final concentration of 10 μg/mL LPS or 1 × 10^5^ CFU/mL *S*. *aureus* ZO3984 or *E*. *coli* VL2874 for 4 h significantly reduced cell viability (Fig 1). Adding 20 μg/mL PLA2G1B to the cells significantly decreased this cell mortality up to 72 h of incubation for LPS and *S*. *aureus* but only 48 h with *E*. *coli* (Fig 1).

**Fig 2:**
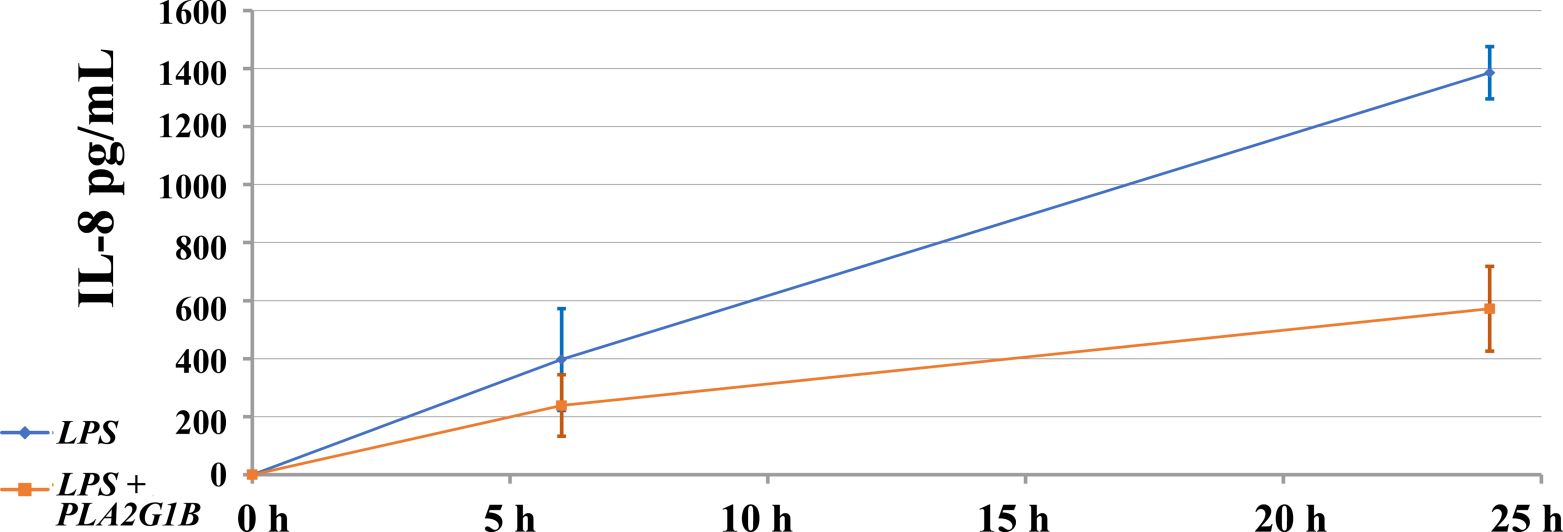
Effect of incubation with PLA2G1B (20 μg/mL) and LPS on IL-8 secretion from bovine mammary epithelial cells. Cells were seeded in 48-well culture plates and IL-8 level in the growth medium was calculated by ELISA test. Standard error bars are presented (n = 8). Cells were simultaneously treated with LPS and PLA2G1B1 and examined at three daily time points.

**Fig 3:**
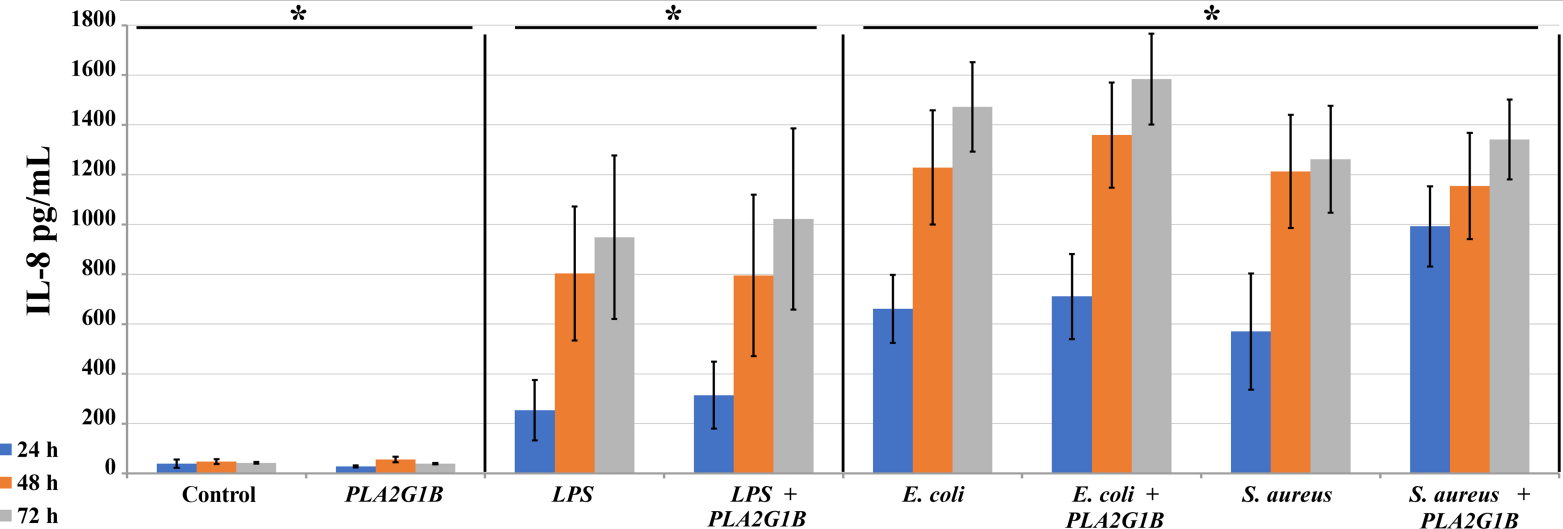
Effect of incubation with PLA2G1B (20 μg/mL) on IL-8 secretion from bovine mammary epithelial cells treated with LPS, *Escherichia coli* or *Staphylococcus aureus*. Cells were seeded in 48-well culture plates and examined at three time points post-treatment; levels of IL-8 in the growth medium were calculated by ELISA test. Standard error bars are presented (n = 6). Cells were first treated with the inflammation agent for 4 h and then incubated in fresh medium with PLA2G1B. Examination time points were 1, 2 and 3 days. Asterisks denote significant differences among the three groups of treatment types denoted by each of the horizontal bars (*P* < 0.05).

When bMECs were co-incubated with 10 μg/mL LPS and 10, 20 or 40 μg/mL PLA2G1B, we observed significant reduction of IL-8 secretion regardless of dose (Fig 2). However, this experimental layout did not follow the sequence of events expected *in vivo*, where the cow is first infected and then treated. To simulate the latter scenario, we first applied the inflammatory agent for 4 h and then replaced the cell medium with fresh medium containing PLA2G1B. Incubation with PLA2G1B of cells that were not treated with any inflammatory agent did not stimulate bMEC secretion of IL-8 or other cytokines (Fig 3, data for TNFα and IL-1β are not shown). Treatment with LPS, *S*. *aureus* or *E*. *coli* for 4 h with or without a following PLA2G1B incubation resulted in continuous IL-8 secretion by bMECs. In cell culture treated with 10 μg/mL LPS, accumulation of 0.25 μg/mL IL-8 was observed after 24 h and the IL-8 level in the medium gradually increased ~4-fold to peak levels at 72 h. A higher rate of IL-8 increase was observed in the medium of cell cultures treated with bacteria, and at all of the tested time points, IL-8 levels were significantly higher for the bacteria than for LPS (Fig 3, *P* < 0.05 by Student’s *t*-test, n = 6). No difference was found in IL-8 secretion between the LPS or bacterial treatments when incubated with PLA2G1B. TNFα was detected only in cells stimulated with *S*. *aureus* with or without PLA2G1B, and no IL-1β secretion was detected (data not shown).

### *In vivo* mammary treatments

#### Experiment 1

**Fig 4:**
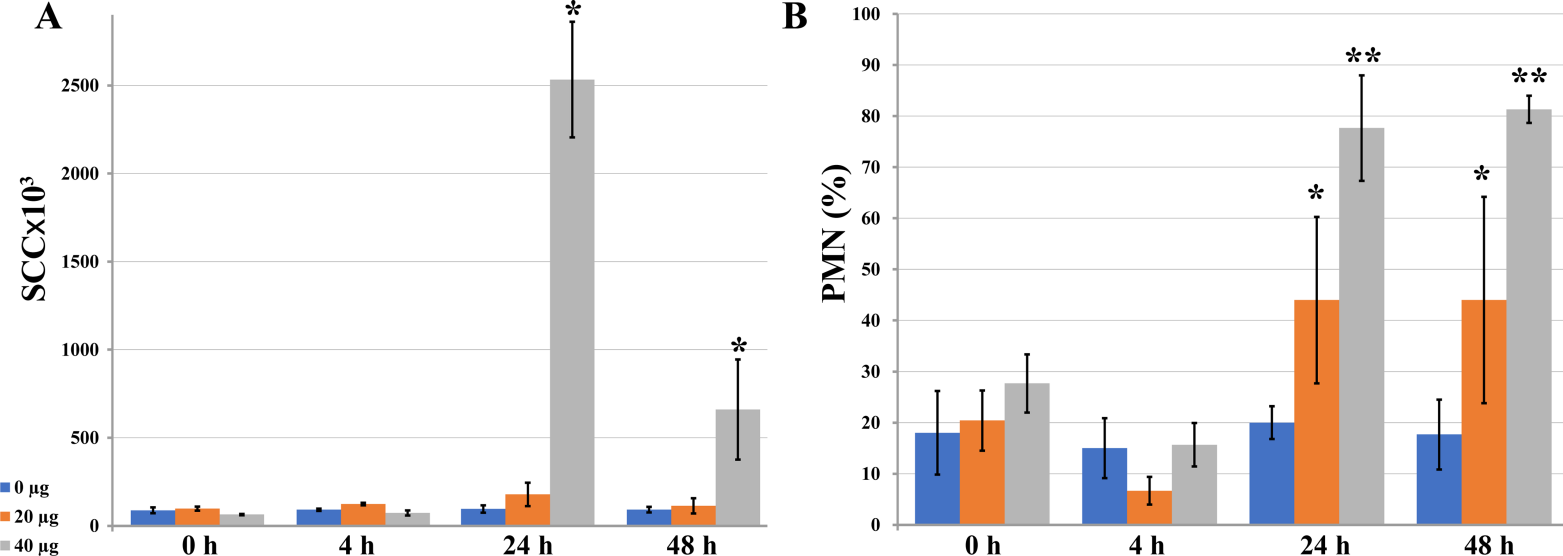
Effects of mammary infusions of PLA2G1B (0, 20, 40 μg) on somatic cell count (SCC) in cow milk. Milk was sampled from mammary glands following mammary infusions of PFS with no phospholipase (control, blue) and with 20 (orange) or 40 (gray) μg PLA2G1B. For the measurements, standard error bars are presented (n = 3). (A) Somatic cells were counted. Asterisks denote significant differences between the denoted and all other measurements (*P* < 0.05, n = 3). (B) Percentage of polymorphonuclear leukocytes (PMN) within the leukocyte population was calculated in each milk sample. Asterisks denotes significant differences between the similarly denoted pairs and the measurements at 4 h after treatment (*P* < 0.05, n = 3).

Initially, we tested the safety of *in vivo* application of PLA2G1B. No symptoms of local inflammation were observed at any of the tested doses in glands infused with PLA2G1B (Fig 4). Control infusions of PFS (without PLA2G1B) did not change the SCC or the percentage of PMNs during the 48-h period. The lower dose of PLA2G1B (20 μg/gland) did not change the total SCC, but there was a significant increase in the percentage of PMNs to ~40% on days 1 and 2 post-infusion. The higher dose of 40 μg/gland caused an increase in SCC on day 1, which decreased on day 2. A sharp increase in PMNs to ~80% was observed on days 1 and 2 (Fig 4). Hence, PLA2G1B induced a mild cellular reaction in the mammary gland but did not elicit clinical signs of inflammation. No effects were observed on milk yield or biochemical composition.

#### Experiment 2

**Fig 5:**
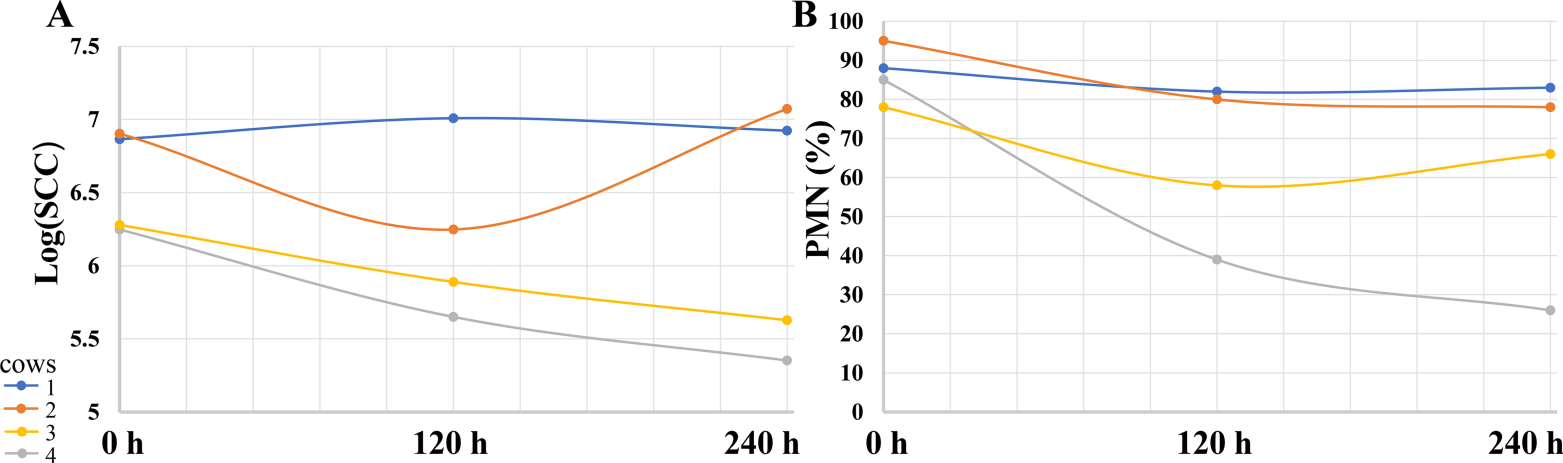
Effects of mammary infusions of PLA2G1B (30 μg) on somatic cell count (SCC) in cow milk of mammary glands with chronic infection due to *Streptococcus dysgalactiae*. Milk was sampled from four mammary glands of four cows (blue, orange, yellow and gray) chronically infected with *S*. *dysgalactiae* following mammary infusions of 30 μg PLA2G1B per quarter. Milk was sampled at three time points (day 0, 5 and 10). (A) Somatic cells were counted. (B) Percentage of polymorphonuclear leukocytes (PMN) within the leukocyte population was calculated in each milk sample.

To examine PLA2G1B as a potential cure for subclinical mastitis, four cows chronically infected with *S*. *dysgalactiae* in one gland were treated with 30 μg PLA2G1B diluted in 20 mL PFS. Only one cow, for which both SCC and PMN decreased, was cleared of the bacteria (Fig 5, gray lines). SCC decreased in 2/4 cows (Fig 5A, yellow and gray lines). Eight cows chronically infected with CNS in one or two glands were treated with 30 μg PLA2G1B. None of these cows were cured of the bacteria and only minor changes were noted in SCC and leukocyte distribution (data not shown).

#### Experiment 3

**Fig 6:**
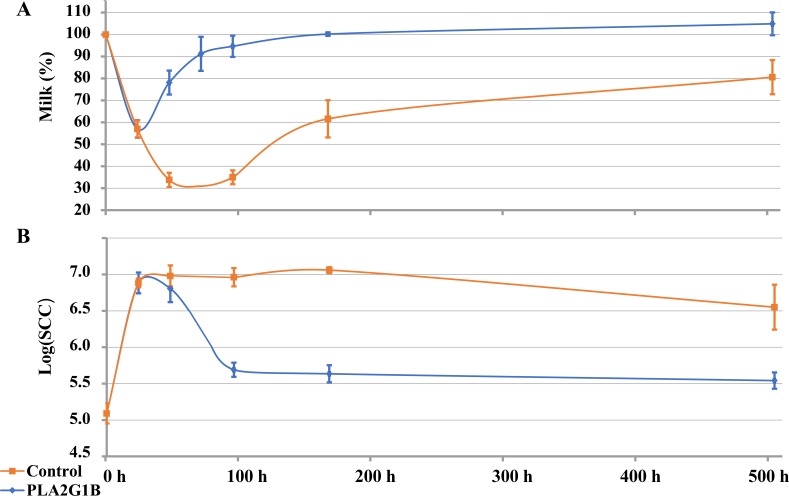
Effects of mammary infusions of PLA2G1B (30 μg) on mammary glands challenged with *Escherichia coli*. Twelve mammary glands of 12 different cows were challenged with *E*. *coli* (strain VL2874, 10–30 CFU). Following mammary infusions of either PFS with no phospholipase (n = 6, control, orange) or with 30 μg PLA2G1B (n = 6, blue), milk was sampled at seven time points (0, 1, 2, 3, 4, 7, 21 days after intramammary infusion). For the measurements, standard error bars are presented (n = 6). (A) Milk yield was measured as percentage of initial yield. (B) Somatic cells were counted (SCC).

To examine PLA2G1B as a potential cure for clinical mastitis, mammary glands were challenged with *E*. *coli* and inoculated with PLA2G1B. The changes in milk yield and SCC for 21 days post-inoculation of treated and control cows are summarized in Fig 6. Of the control cows, two out of six were cleared of bacteria between days 5 and 7, whereas in the other four cows, the bacteria were still there on day 21 (end of the experiment). In contrast, in the treated cows, *E*. *coli* was not detected in milk of four out of six cows on day 5, and of six out of six cows on day 7. In all cows, the percentage of milk yield out of yield on day zero (100%) was depressed by ~40% on the first day before treatment with PLA2G1B. In the control glands, the milk yield continued to decline by ~70% between days 2 and 4, and on day 21 it was back to a level ~20% lower than that of day zero (Fig 6A). Of the treated cows, 1 day after treatment, the milk yield started to increase, approaching the level recorded before inoculation (90–100%) on day 5. One day after inoculation with *E*. *coli*, SCC increased in all cows by 63-fold to ~7.9 × 10^6^ cell/mL (Fig 6B). In the control cows, SCC remained at this high level up to day 7 post-inoculation and on day 21 was still an average of 25-fold higher than that of time zero. In contrast, SCC of the treated cows decreased on day 4 to 4.9 × 10^5^ cell/mL and returned to the level before inoculation on day 7 (Fig 6B).

## Discussion

In a murine model of mastitis, it has been shown that intramammary infusion of bovine PLA2G1B to the infected gland relieves visible and histological inflammation and reduces blood levels of infiltrating lactose [20]. Following this and other indications that sPLA2s have primarily an anti-inflammatory role in the inflammatory response [16], we investigated the application of PLA2G1B as a therapeutic for bovine mastitis. To minimize the risk of inflicting tissue damage with this digestive enzyme, we first applied it to *in vitro*-cultured cells, and did not observe any changes in viability or in the pattern of secretion of TNFα, IL-1β or IL-8 cytokines. However, when the cell culture was treated with LPS or live bacteria, incubation with PLA2G1B significantly improved cell viability. As both *in vivo* and in cultured cells, inflammation agents such as LPS stimulate the generation of mitochondrial reactive oxygen species and the accumulation of oxidative damage to the cell [31,32], it may be that the mechanism involved in the observed improved viability of bMECs was PLA2 protection of membranes from lipid-peroxidation damage [10,17]. Of the four modes of action for resolving sPLA2s, detailed in the Introduction, this explanation (mode (i)) is most plausible as in culture media, PLA2G1B cannot neutralize LPS, it has virtually no bactericidal action (mode (ii)) [20] and there are no macrophages to attract (mode (iii)). Mimicking the natural sequence of events, PLA2G1B was applied after challenge with the inflammation agent, and no effect on cytokine secretion from bMECs was observed, making involvement of cellular transduction (mode (iv)) less likely. However, when PLA2G1B was applied simultaneously with LPS, a significant short-term reduction in IL-8 secretion was observed compared to bMECs that were only treated with LPS. A significant effect on mRNA expression of this neutrophil chemotactic factor was also observed in a very similar system, where the response of bMECs to co-incubation of PLA2G1B and LPS was tested [33]. In that system, following LPS challenge, bMECs that were not treated with phospholipase exhibited significant downregulation of IL-8 secretion compared to bMECs that were treated with both LPS and PLA2G1B. Since endothelial cells pre-store IL-8 in their Weibel–Palade bodies [34], the release of this cytokine is not expected to be directly coupled with its mRNA levels, which may reflect the cell’s regulation in replenishing or reducing these stores. The exogenous administration of PLA2G1B to bMECs revealed altered expression of several pro-inflammatory genes in response to LPS [33]. Hence, combining the conclusion of the latter research group that highlights PLA2G1B as a therapeutic candidate, with our findings that *in vitro*, this phospholipase improves cell survival under stress mediated by inflammation factors, we concluded that its *in vivo* examination in the bovine mammary gland was feasible.

The examination of a single PLA2G1B treatment as a potential cure for subclinical mastitis in cows chronically infected with *S*. *dysgalactiae* consisted of a small sample size. This bacterium is listed within the main mastitis causing pathogens with biofilm formation ability [4]. Although spontaneous cure from infection with *S*. *dysgalactiae* was reported [35], spontaneous remission from a chronic state, which involves formation of a resistant biofilm, has never been recorded. Thus, this observation of a cure rate of 1/4 should be further investigated. Further experimentation on a larger sample is needed to validate this finding and to develop a protocol for application of PLA2G1B as a treatment for subclinical mastitis. It is possible that the typical ability of sPLA2s to aggregate with cellular debris and facilitate their internalization by macrophages (mode (iii)) [16] played a role in eliminating this bacterial biofilm.

A single mammary infusion of PLA2G1B to the bovine model of clinical mastitis demonstrated significant relief from disease symptoms. This examination was based on introducing *E*. *coli* via the streak canal and treating the challenged gland by phospholipase infusion, after recording the clinical symptoms. In this case, it is not obvious which of the four modes of resolving inflammation by sPLA2s was dominant. Milk quality and yield may remain affected for a long period of time following an episode of *E*. *coli* mastitis, regardless of bacterial clearance in the gland [36]. In such cases, antibiotic treatments are useless and novel therapeutics that do not require discarding milk and that promote the recovery of the affected glands are required. As treatment with PLA2G1B proved harmless to cultured cells, we believe that following treatment, no withdrawal time is required and thus there would be milk save for consumers if mastitis would be treatment with this phospholipase. Overall, the presented results strongly support further study of PLA2G1B as a remedy for some forms of bovine mastitis.

## Supporting information

S1 File ARRIVE guidelines checklist. A checklist for reporting in vivo experiments of this animal research (experiments 1–3).
(PDF)
